# SOXE transcription factors form selective dimers on non-compact DNA motifs through multifaceted interactions between dimerization and high-mobility group domains

**DOI:** 10.1038/srep10398

**Published:** 2015-05-27

**Authors:** Yong-Heng Huang, Aleksander Jankowski, Kathryn S. E. Cheah, Shyam Prabhakar, Ralf Jauch

**Affiliations:** 1 Genome Regulation Laboratory, Guangzhou Institutes of Biomedicine and Health, 190 Kai Yuan Avenue,Science Park, 510530 Guangzhou, China; 2 Computational and Systems Biology, Genome Institute of Singapore, Singapore 138672, Singapore; 3 Faculty of Mathematics, Informatics and Mechanics, University of Warsaw, Banacha 2, 02-097 Warszawa, Poland; 4 Department of Biochemistry, Li Ka Shing Faculty of Medicine, The University of Hong Kong, 21 Sassoon Rd, Hong Kong, China

## Abstract

The SOXE transcription factors SOX8, SOX9 and SOX10 are master regulators of mammalian development directing sex determination, gliogenesis, pancreas specification and neural crest development. We identified a set of palindromic SOX binding sites specifically enriched in regulatory regions of melanoma cells. SOXE proteins homodimerize on these sequences with high cooperativity. In contrast to other transcription factor dimers, which are typically rigidly spaced, SOXE group proteins can bind cooperatively at a wide range of dimer spacings. Using truncated forms of SOXE proteins, we show that a single dimerization (DIM) domain, that precedes the DNA binding high mobility group (HMG) domain, is sufficient for dimer formation, suggesting that DIM : HMG rather than DIM:DIM interactions mediate the dimerization. All SOXE members can also heterodimerize in this fashion, whereas SOXE heterodimers with SOX2, SOX4, SOX6 and SOX18 are not supported. We propose a structural model where SOXE-specific intramolecular DIM:HMG interactions are allosterically communicated to the HMG of juxtaposed molecules. Collectively, SOXE factors evolved a unique mode to combinatorially regulate their target genes that relies on a multifaceted interplay between the HMG and DIM domains. This property potentially extends further the diversity of target genes and cell-specific functions that are regulated by SOXE proteins.

The SOX (SRY-related HMG box) gene family of transcription factors (TFs) comprises 20 members in human and mouse genomes. SOX genes regulate stemness, direct cellular identities and demarcate developmental domains^1^,^2^,^3^. All family members share a 79 amino acid high-mobility-group (HMG) box domain that adopts an L-shaped structure with major and minor wings made up of three alpha-helices and aligned N and C-terminal extensions^4^,^5^,^6^,^7^,^8^,^9^. The HMG domain mediates the selective recognition of a CATTGT-like sequence by docking to the minor groove of the DNA. The binding leads to a sharp DNA kinking to around 70 °C induced by the intercalation of a Phe-Met dipeptide into the central ‘TT’ basepair and asymmetric neutralization of the negatively charged phosphate backbone by the positively charged tails of the HMG box^10^. Based on the primary amino acid sequence, paralogous members were further subdivided into 8 subgroups denoted SOXA to SOXH^11^. The SOXE group comprises three members termed SOX8, SOX9 and SOX10^12^. SOXE proteins are expressed in many cell types and function pleiotropically to direct diverse biological processes including chondrogenesis, gliogenesis, sex determination, pancreatic development, skin development and kidney development^13^,^14^,^15^,^16^,^17^. Apparently, SOXE function is highly context dependent and SOXE proteins bind to and regulate different sets of genes in different cellular environments. Moreover, SOXE factors play critical roles in stem cell biology and cellular reprogramming. For example, SOX9 is part of a cocktail facilitating the conversion of fibroblasts into sertoli-like cells^18^ and chondrocytes^19^,^20^. SOX9 also induces and maintains neural stem cells^21^. Further, SOX10 regulates stemness and multipotency in neural crest stem cells^22^ and enables the induction of multipotent neural crest cells when singly expressed in human fibroblasts^23^.

SOXE loss-of-function mutations are associated with many human diseases. For example, heterozygous SOX9 mutations cause campomelic dysplasia (CD), a skeletal malformation syndrome with autosomal sex reversal^24^,^25^. A unique feature of the SOXE group is a 40 amino acid ‘DIM’ region N-terminally preceding the HMG domain that mediates DNA dependent homodimerization^26^,^27^. The structure and the mechanism of how the DIM mediates dimerization are unknown. Interestingly, some mutations leading to the CD phenotype are single non-sense mutations within the DIM^28^,^29^. The context dependent dimerizations of SOX9 have furthermore been proposed to set apart two contrasting developmental roles of this protein. According to this model, dimeric SOX9 is required for a gene expression program leading to chondrogenesis whilst monomeric SOX9 regulates sex determination^28^. Likewise, mutant mice expressing a dimerization incompetent SOX10 with a triple alanine mutation in the DIM domain showed highly context dependent abnormalities suggesting that dimerization is critical for some but not all SOX10 mediated developmental processes^30^.

Given the relevance of SOXE dimerization for human disease and its potential to determine the cell-specific roles of SOXE factors, we set out to interrogate the basis for homo- and heterodimerisation of SOXE proteins using quantitative electrophoretic mobility shift assays. We found that SOXE factors can effectively bind to a range of composite DNA elements with flexible half-site spacing enriched in the enhancers of melanoma cell lines. All SOXE factors SOX8, SOX9, SOX10 can also cooperatively heterodimerize. In particular we found that one DIM domain suffices for dimer formation indicating that the dimerization is driven by DIM:HMG rather than DIM:DIM interactions. This SOXE HMG property is specific to SOXE proteins as the HMG boxes of SOX4, SOX2, SOX6 and SOX18 lack the ability to cooperatively dimerize. The SOXE proteins have important functions in a wide range of cell types- for example chondrocytes, neural progenitors, otic cells, sertoli cells, oligodendrocytes and glial cells. Our data implicate that direct combinatorial partnerships amongst SOXE factors, mediated by both intramolecular as well as intermolecular interactions between DIM and HMG domains, occurring on a range of composite DNA motifs, could direct such diverse cellular identities.

## Results

### SOX dimer motifs are enriched in regulatory regions specific to melanoma cells

**Figure 1:**
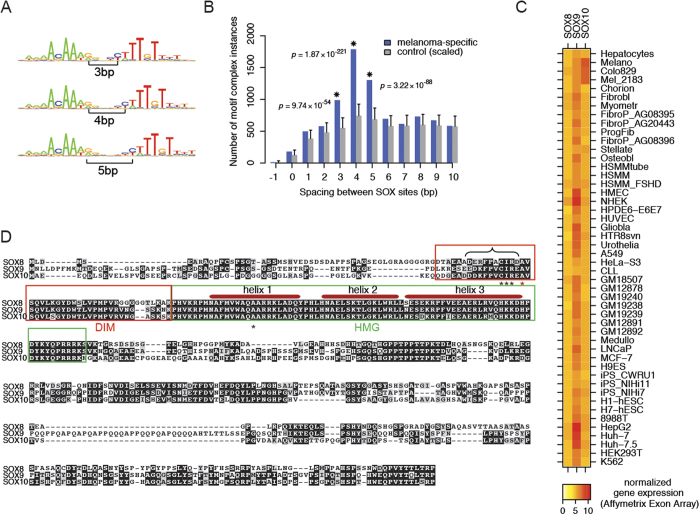
Melanoma cell enhancers are enriched for certain palindromic SOX motifs, suggesting SOXE dimerization. (**A**) Palindromic composite SOX motifs, consisting of juxtaposed TRANSFAC motifs, found overrepresented in melanoma-specific DNase I hypersensitive sites (DHS). Spacer size is indicated. (**B**) Numbers of instances of palindromic SOX complexes within melanoma-specific DHS (blue bars) for various spacings between SOX motifs. Grey bars indicate the expectation based on the control set, along with significance threshold for *p* < 0.05 after Bonferroni correction. Asterisks indicate strongly overrepresented SOX dimers; their Bonferroni-corrected *p*-values calculated by TACO^31^ are shown. (**C**) ENCODE/Duke Affymetrix Exon Array expression levels for human SOXE genes in 48 of the 59 cell lines used for dimer motif detection. For the remaining 11 cell lines such expression data were not available. SOX10 shows an expression peak in melanoma cell lines (Colo829, Mel_2183 and Melano). (**D**) Alignment of mouse SOXE proteins. Black asterisk indicate amino acids shown to be critical for SOXE dimerization^45^. The red asterisk mark the A76E missense mutation and the curly bracket the 66–75 aa deletion mutant detected in campomelic dysplasia patients reported to abrogate cooperative dimer formation^29^. DIM and HMG domains are marked with red or green boxes.

We have recently developed a computational method that detects overrepresented dimer motif configurations in cell-type–specific open chromatin regions as defined by DNase I hypersensitivity (DHS)^31^,^32^. Using this strategy, we identified three palindromic SOX dimer motifs enriched in regulatory regions specific to two human melanoma cell lines, but not in the 57 other ENCODE cell lines used for this analysis (Fig. 1A,B). The most abundant version of the SOX dimer motif was ACAAAG*nnnn*CTTTGT (where the ‘*n’*s constitute a 4 base-pair spacer between the SOX half-sites). This dimer motif was followed by overrepresented variants thereof with 3 and 5 base-pair spacers.

We surmise that SOXE proteins are specifically recruited to these dimer motifs for several reasons. First, SOXE factors are known to dimerize on palindromic composite motifs^26^,^27^,^29^. Second, when inspecting Affymetrix Exon Array expression data from ENCODE/Duke, we found SOX10 to be specifically upregulated in melanoma cell lines (Fig. 1C). Thus, the differential expression of SOX10 in melanoma cells and its preference for dimer motifs in those cells could be causative for the formation of melanoma-specific DHS regions. Furthermore, a motif reminiscent to the 4 bp spacer motif was recently discovered in a SOX9 chromatin immunoprecipitation followed high throughput sequencing (ChIP-seq) study in chondrocytes^33^. However, the motif choice of SOXE factors appears to be cell-type–specific, as SOX9 ChIP-seq in primary hair follicle stem cells did not reveal an enrichment of palindromic sequences^34^. Therefore, cell lines with elevated SOX9 expression such as the liver carcinoma cell lines HepG2 (Fig. 1C) may not contain palindromic SOX dimer elements within their DHS because SOX9 utilizes a different motif configuration to engage the chromatin in those cells. Since SOX10 is a key driver of neural crest development leading to melanocyte specification^15^, as well as pathologic melanoma progression^35^, we decided to biochemically interrogate DNA motif preferences and dimerization patterns of SOXE proteins.

### The SOXE DIM domain mediates cooperative assembly on flexibly spaced dimer motifs

**Figure 2:**
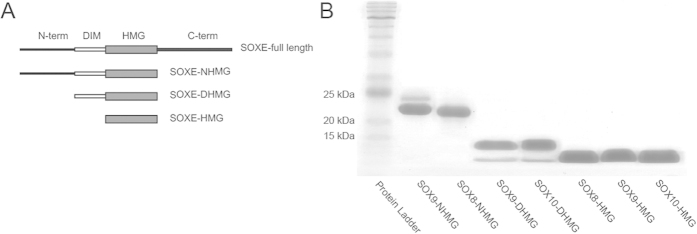
SOXE protein constructs used in this study. (**A**) Diagrams of the protein constructs used for the binding assays. The DIM domain is shown in white and the 79aa HMG domain in gray. (**B**) 15% SDS-PAGE of the purified proteins.

**Figure 3:**
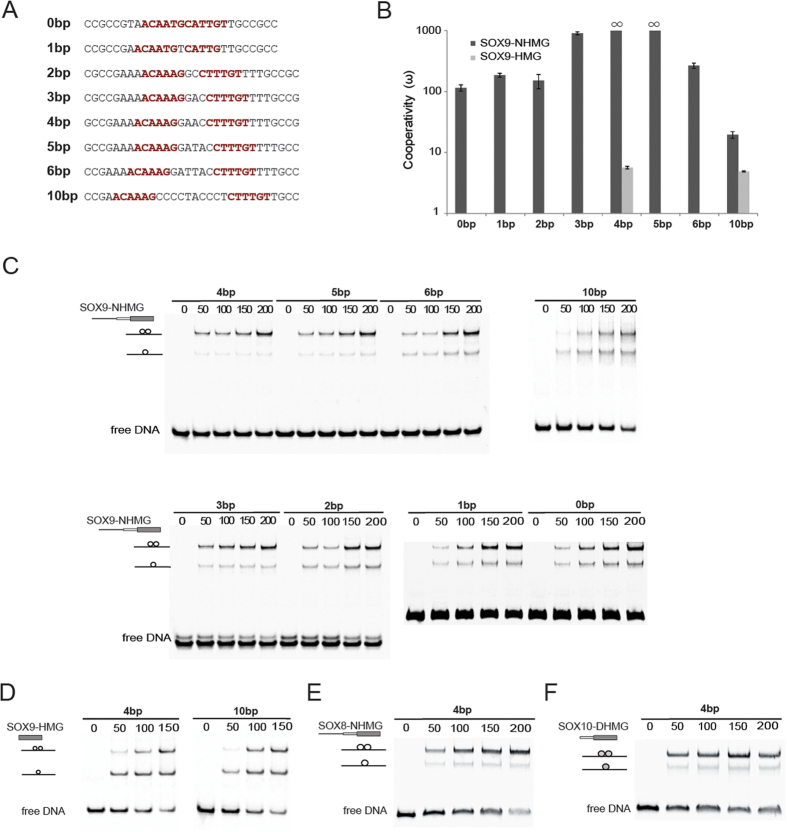
SOXE proteins cooperatively dimerize on flexibly spaced dimer motifs. (**A**) Sequences of the dsDNA used for EMSAs with SOX half-site in bold face. (**B**) Barplots showing the mean cooperativity factors (ω) obtained from 3–6 measurements for the the SOX9-NHMG (black) and SOX9-HMG (gray). ω values were calculated as described^36^. For the 4 and 5bp spacers ω was denoted as infinite as it could not be reliable determined since the fractional contribution of monomer bands is lower than 2% per lane. (**C**) Representative EMSAs of SOX9-NHMG constructs on differently spaced dimer motifs. (**D**) EMSAs of SOX9-HMG constructs on 4 and 10 bp elements. (**E**,**F**) EMSAs of SOX8-NHMG (**E**) and SOX10-DHMG (**F**) constructs on the 4 bp spacer element.Protein concentrations in nM per lane are indicated and construct and ternary complexes are shown with cartoons to the left of the gel image where lines indicate the DNA and circles the proteins.

To test whether SOXE proteins can cooperatively assemble on the melanoma-specific sequence signatures, we conducted electrophoretic mobility shift assays (EMSAs) to quantify the cooperativity factor (ω) using previously described methods^36^. If the value for ω is>1 we will henceforth refer to ‘cooperative’ binding, if it is ~1 to an additive and if smaller than 1 to a competitive binding mode. To conduct these experiments, we constructed a series of SOXE protein variants and purified them to>95% purity as judged by SDS-PAGE (Fig. 2A,B). These comprise SOXE-HMG constructs consisting of the 79 amino acid DNA binding HMG domain; SOXE-DHMG constructs that contains the 40 amino acid DIM domain preceding the HMG domain and a SOXE-NHMG construct that resembles the a naturally occurring truncation mutant^37^ that spans N-terminus, DIM and HMG domains (Figs. 1D, 2A,B). We designed EMSA probes corresponding to the three motifs enriched in melanoma cell enhancers as well as for DNA elements with more distant or more compressed half-site spacing (Fig. 3A). EMSAs demonstrated that SOX9-NHMG binds to the three elements enriched in melanoma cell DHS in a highly cooperative fashion (Fig. 3B,C). By contrast, experiments using the isolated SOX9 HMG domain shows a marked reduction of the cooperativity reaffirming that the DIM domain facilitates the cooperative assembly (Fig. 3B,D). When control motif configurations that are not enriched in melanoma enhancers (0,1,2 bp and 6 bp) were tested, a ~10-fold diminished cooperativity factor was measured (Fig. 3B,C). Nevertheless, with a cooperativity factor of ~100, dimers are still formed efficiently. Only the 10 bp spacer led to a more pronounced drop of the cooperative binding (Fig. 3B,C). The SOX8-NHMG and SOX10-DHMG constructs cooperate on the 4 bp element equally strong as the SOX9-NHMG suggesting that the cooperative recognition of palindromic DNA elements is a conserved property shared by all SOXE TFs (Fig. 3E,F).

### SOX8, SOX9 and SOX10 form cooperative heterodimers mediated by a single DIM domain

**Figure 4:**
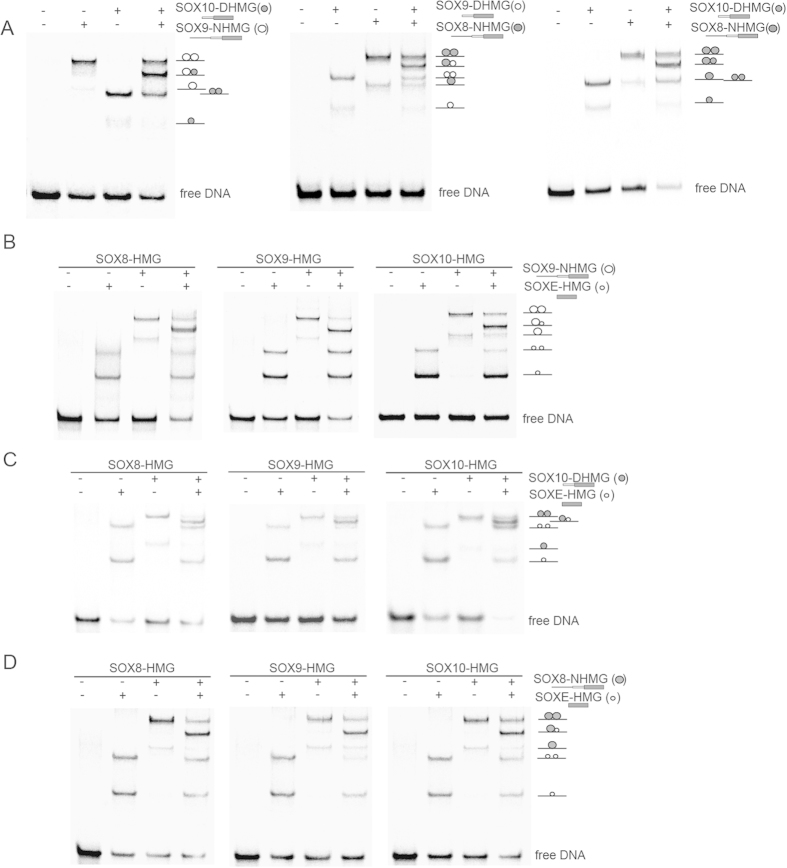
Cooperative SOX8/9/10 heterodimerization is promoted by a single DIM domain. (**A**) EMSAs after mixing DIM containing SOXE proteins lead to the formation of a prominent heterodimer band in addition to homodimer bands. (**B**) Mixing of the SOX9-NHMG with the isolated HMGs of SOX8, SOX9 and SOX10 results in the formation of SOXE-HMG:SOX9-NHMG heterodimers that are more abundant than the SOX9-NHMG homodimers or SOXE-HMG homodimers. (**C**,**D**) Likewise, when the SOX9-NHMG was replaced by the SOX10-DHMG (**C**) or SOX8-NHMG (**D**) constructs to monitor heterodimer formation the SOX10-DHMG:SOXE-HMG and SOX8-NHMG:SOXE-HMG heterodimers were found to be the most prominent complexes.

SOXE proteins can be expressed singly or in different combinations in several cell types but in many instances function non-redundantly^15^,^17^. As transcription factors (TFs) in general, and SOX factors in particular, act combinatorially and switch partners to perform cell-type specific functions^38^, we decided to study SOXE heterodimerization. Although the HMG as well as the DIM domain is highly conserved between SOX8, SOX9 and SOX10, there are several amino acid substitutions that could cause discriminatory complex formation leading to paralog specific functions (Fig. 1D). Importantly, rather subtle missense mutations within the SOXE-DIM are associated with the CD phenotype^28^,^29^ (Fig. 1D). To be able to distinguish SOXE homodimers and heterodimers in EMSAs, we used protein constructs of different lengths (Fig. 2A,B). When we mixed SOXE constructs with the 4bp spacer element, we found that SOX9-NHMG:SOX10-DHMG, SOX9-DHMG:SOX8-NHMG or SOX8-NHMG:SOX10-DHMG heterodimers migrate as clearly distinguishable bands in EMSAs (Fig. 4A). Moreover, heterodimer bands are as prominent as the respective homodimer bands suggesting that heterodimerization between SOXE pairs occur in a highly cooperative manner (Fig. 4A). This result is consistent with a previous study reporting SOX8-SOX10 heterodimers^39^.

Several TF families harbor dimerization or multimerization domains including the Smad family^40^, SCAN C2H2 zinc fingers^41^ and nuclear receptors (NRs)^42^. In those cases, the dimerization (SCAN-C2H2 TFs and NRs) or trimerization (Smads) is mediated by reciprocal interactions between the multimerization domains (mad homology 2 (MH2) for Smads, the SCAN domain for SCAN-C2H2 zinc fingers, and the ligand binding domain (LBD) for NRs). We therefore assumed that the DIM of SOXE promotes dimerization predominantly through DIM:DIM interactions. However, to our surprise, when we mixed DIM containing SOX9-NHMG constructs with isolated SOX8-HMG, SOX9-HMG and SOX10-HMG proteins, we observed distinctive SOX9-NHMG:SOXE-HMG complexes (Fig. 4B). In fact, SOX9-NHMG:SOXE-HMG complexes migrate as more prominent bands than homodimeric SOX9-NHMG:SOX9-NHMG complexes suggesting the preferential formation of SOX9-NHMG:SOXE-HMG heterodimers (Fig. 4B). Similar observations were made when the SOX10-DHMG or SOX8-NHMG constructs were mixed with SOX8-HMG, SOX9-HMG or SOX10-HMG constructs, respectively (Fig. 4C,D). Therefore, a single DIM domain is sufficient to support cooperative dimer formation between SOXE TFs suggesting that DIM:HMG rather than DIM:DIM interactions facilitate cooperative DNA recognition.

### The HMG box mediates selective dimerization of SOXE factors

**Figure 5:**
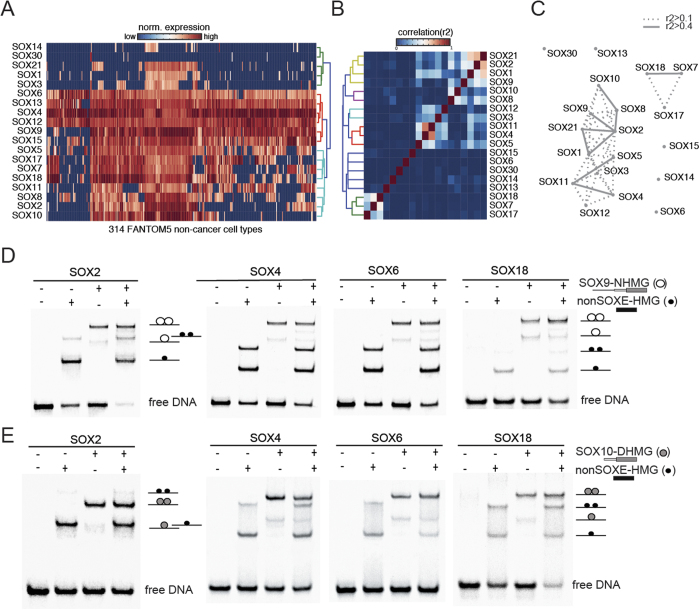
The SOXE-HMG encodes selectivity determinants (**A**) Expression data for human SOX proteins reported by the FANTOM5 consortium^14^ was hierarchically clustered in glbase^60^ showing the widespread co-expression of SOX proteins in many cell types. Expression data were transformed into a correlation heatmap (**B**) or into a network using r2 correlation coefficients based on the co-expression similarity of SOX genes (**C**) further illustrating the frequent co-occurrence of SOXE factors (in particular SOX8 and SOX10) but also highlights the correlation of SOXE factors with SOXB family members SOX1, SOX2 and SOX21. Network nodes are SOX TFs and edges are drawn between nodes if the r2 correlation across the FANTOM5 expression dataset is >0.4 (bold lines) or >0.1 (dotted lines). (**D**,**E**) The SOX9-NHMG (**D**) or the SOX10-DHMG (**E**) was mixed with the HMG boxes of SOX2, SOX4, SOX6 and SOX18 to assess whether SOXE proteins have the capacity to interact with non-SOXE factors. SOX9-NHMG/non-SOXE-HMG heterodimers are barely visible and the SOX9-NHMG or SOX10-DHMG homodimers and non-SOXE-HMG monomers pre-dominate. Non-SOXE-HMG boxes are indicated using black filled circles and the SOX9-NHMG as white circles and the SOX10-DHMG as gray circles in the cartoons marking the microstates to the right of the gel images. A 109aa HMG construct was used for SOX2 and 79aa HMG constructs for SOX4, SOX6 and SOX18 explaining the different mobility.

The finding that only one DIM domain is necessary to mediate effective DNA dependent dimerization with HMG boxes raises the intriguing question as to whether SOXE proteins cooperate with other members of the SOX family. To assess whether such interactions are in principle possible, we first asked whether SOXE factors are co-expressed with non-SOXE TFs. To this end we inspected the expression of all 20 SOX proteins in 315 primary human tissues as reported by the FANTOM5 consortium^14^. We found that SOX9 is broadly expressed in the majority of the 315 cell types while the expression domains of SOX8 and SOX10 are slightly more restricted (Fig. 5A). As most other SOX factors (exceptions being SOX14 and SOX30) show an equally widespread tissue distribution, SOXE factors are co-expressed with most non-SOXE in some cell types giving rise to a plethora of theoretically possible heterodimer combinations (Fig. 5A). When the expression data are clustered in a correlation heatmap (Fig. 5B) or represented as a network based on expression correlation (Fig. 5C), we found that the three SOXE TFs frequently co-occur but they also cluster with the SOXB group proteins SOX1, SOX2 and SOX21. Given the prevalent co-expression of SOXE and non-SOXE TFs, we decided to test whether SOXE factors can heterodimerize with isolated HMG boxes of SOX2 (SOXB), SOX4 (SOXC), SOX6 (SOXD) and SOX18 (SOXF) representing the major subgroups of the SOX family. However, SOX9-NHMG (Fig. 5D) and SOX10-DHMG (Fig. 5E) homodimers predominate on all EMSAs containing SOXE/non-SOXE-HMG mixtures and SOXE/non-SOXE-HMG heterodimers are barely detectable. Rather, the HMG boxes of SOX2, SOX6, SOX4 and SOX18 form prominent monomeric complex or weakly homodimerize. Collectively, these results show that SOXE factors evolved a unique protein module, the DIM domain, accompanied by co-evolving HMG box features to promote selective dimerization.

### Intra and intermolecular DIM:HMG interactions promote SOXE dimerization

**Figure 6:**
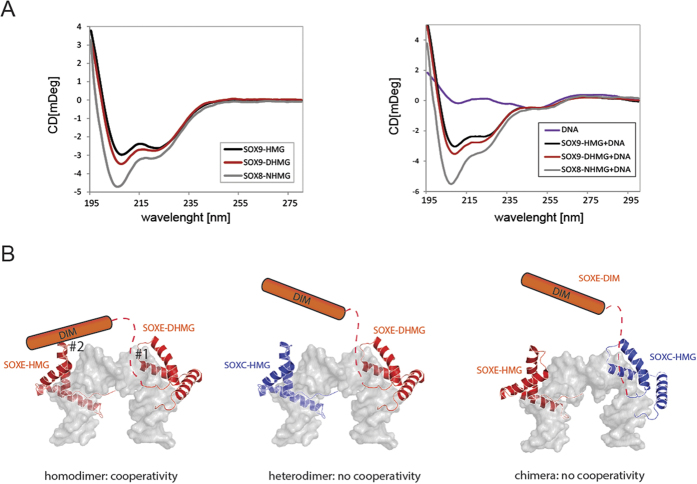
Model for the multifaceted interactions that mediate dimerization of SOXE proteins on DNA (**A**) Circular dichroism spectra of various SOXE protein constructs in the absence (left panel) or presence (right panel) of DNA. (**B**) Models were built using the Tfam structure (PDB id 3TMM)^43^ and the SOX9 structure (PDB id 4EUW) as templates. The DIM domain is depicted with a dashed line and a red box. SOXE HMGs are shown in red and non-SOXE HMGs in blue. For simplicity no more than one DIM domain is shown in the left panel. We propose an intramolecular interaction between the dashed SOXE linker with the SOXE-HMG (#1) which is communicated to the DIM which in turn engages intermolecularly the HMG of the neighboring SOXE-HMG (#2) leading to cooperative dimerization. If the neighboring molecule is a non-SOXE-HMG the intramolecular interaction is not supported. Likewise, a non-SOXE-HMG would not support the intramolecular linker-HMG interaction as implied by EMSAs using chimeric SOXE-DIM/non-SOXE-HMG proteins^45^.

We next investigated the structural basis for the SOXE dimerization. We first recorded circular dichroism spectra of SOXE-NHMG, SOXE-DHMG and SOXE-HMG proteins. As expected from published crystal structures, the SOX9-HMG shows a spectrum indicative of an α-helical protein with peaks at 208 and 222 nm (Fig. 6A). The SOX9-DHMG spectrum shows only a slight increase of the peak at 208 while the SOX8-NHMG spectrum shows a more pronounced peak at this wavelength (Fig. 6A). This suggests some additional helical structures at the N-terminus. DNA addition leads to only minor changes in the CD spectrum suggesting that the secondary structures are likely pre-formed and not induced by protein-DNA or protein-protein interactions. The crystal structure of the DNA bound tandem HMG box of the transcription factor A (Tfam) has previously been shown to induce a U-turn to promoter DNA^43^. While Tfam belongs to the class of non-sequence specific HMG boxes in contrast to the sequence specific SOX family, the overall fold and the mechanism of bending is similar in both classes^44^. We therefore used the Tfam structure (PDB id 3tmm) as a template to model a SOX9 homodimer (Fig. 6B). As the structure of the DIM is unknown, the model only consists of the HMG domains. Helices 1 and 2 of the minor wing face each other while helix 3 and the major wing occupy rather remote positions. Consistently, helices 1 and 2 were previously shown to be critical for dimer formation while helix 3 was interchangeable between SOXE and non-SOXE HMGs^45^. Therefore, in contrast to SOX-OCT interactions that critically rely on interfaces presented by helix 3 of the HMG^8^,^46^,^47^, the SOXE-HMG likely utilizes an alternative interface to interact with the DIM domain.

Previous studies suggested a reciprocal interaction between HMG:HMG and DIM:DIM domains^45^. However, we suggest an intramolecular DIM : HMG interaction that allosterically positions the DIM domain for a further DIM:HMG interaction. We suggest this model because: (i) isolated HMG domains do not cooperate suggesting a paucity of direct HMG:HMG interactions; (ii) a single DIM domain is sufficient to support effective DHMG:HMG dimerization indicating that DIM:DIM interactions are not necessary. Rather DIM:HMG interactions mediate dimerization; (iii) the isolated SOXE-HMG does not cooperate with a chimera consisting of a SOXE-DIM and a non-SOXE-HMG suggesting that intermolecular DIM:HMG interactions are not sufficient to facilitate cooperativity^45^. The latter observation points towards a peculiar cross-talk between molecular interfaces (denoted #1 and 2# in Fig. 6B). Considering that cooperative dimers can be formed on a range of flexible motif configurations, the most parsimonious explanation would have been an intermolecular DIM:HMG interaction supported by a structurally flexible linker. However, in such a scenario the SOXE-DIM/non-SOXE-HMG chimera would be expected to cooperate as effectively with a SOXE-HMG as non-chimeric SOXE-DHMG constructs (Fig. 6B). It cannot be completely ruled out that the cooperativity is communicated indirectly by an allosteric, DNA mediated mechanism rather than by direct protein-protein interactions that are formed as a consequence of DNA recognition. However, given the size of the DIM domain, the juxtaposition of the interfaces in structural models and the detrimental effect of HMG mutations remote from the protein-DNA interaction surface, we expect direct protein-protein interactions in the context of appropriately configured composite DNA binding sites to be the main driver for cooperative complex formation. Nevertheless, the precise mechanism of how the intramolecular cross-talk between DIM and HMG domains is propagated to the intermolecular interface would benefit from structural characterization.

## Discussion

Many TF dimers cooperatively associate on highly compact and constrained composite DNA elements^32^,^48^,^49^. By contrast, SOXE proteins possess the capacity to retain substantial cooperativity on a wide range of flexibly spaced composite DNA motifs. We previously predicted that TFs whose dimerization was mediated by contacts between DNA-binding domains would tend to bind tightly juxtaposed half-sites, and moreover such dimers would not tolerate changes in the half-site spacing. In contrast, if other domains mediated dimerization, half-site spacing would be wider and some degree of variability in half-site spacing may be tolerated^31^,^32^. SOXE dimerization, which is mediated by the non-DNA-binding DIM domain, appears to provide an example of the latter.

Does dimer formation endow SOXE proteins with unique functional properties? SOX9 dimerization was demonstrated to be necessary for chromatin remodeling^50^. Similarly, SOX2, one of the key pluripotency reprogramming factors, was found to acquire chromatin opening ‘pioneering’ ability after forming a complex with OCT4 and KLF4^51^. However, the genome-wide modeling of DHS data has suggested that monomeric SOX proteins do not possess pioneering activity^52^. Therefore, SOX-mediated formation of TF complexes in general and SOXE dimerization in particular could influence their pioneering activity. Moreover, genome-wide binding studies revealed that SOX proteins target only a small subset of high-affinity binding sites encoded in mammalian genomes^53^,^54^,^55^. Even in the context of accessible ‘open’ chromatin the presence of high affinity SOX binding sites is not predictive for binding^52^. Evidently, SOX proteins require additional directives from partner proteins to select their genomic target sites^11^,^38^,^56^. There are several well-described heterologous partner factors from non-SOX TF families such as POU and PAX family proteins^8^,^48^. It will be interesting to further explore whether and how SOXE dimerization affects the selection of genomic loci in open chromatin and how it influences their pioneering activity. While SOXE dimerization could simply enhance a transcriptional response by forming a more stable and longer lasting complex^27^, it is conceivable that SOX monomers and the various homo- and heterodimeric complexes lead to qualitatively different responses. For example, a SOX9 homodimer could elicit a different outcome than a SOX9/SOX10 heterodimers by recruiting different co-factors such as chromatin remodelers or enzymes catalyzing epigenetic modifications.

## Materials and Methods

### Computational analysis of SOX dimer motifs

We used our previously developed tool, TACO^31^, to identify overrepresented SOX dimer motifs. We applied our method to a comprehensive collection of DNase-seq datasets from Duke University (Genome Browser track wgEncodeOpenChromDnase) provided by the ENCODE Project Consortium^57^. Datasets for 59 untreated cell lines were clustered according to their similarity, resulting in 26 cell types, as described previously. In particular, two melanoma cell lines (Colo829 and Mel_2183) were considered as a single cell type. To identify motif complex enrichment, we considered the cell-type–specific portions of DNase I hypersensitive sites for each cell type and compared them against the union of all DNase I datasets (control set). All the pairs of SOX motifs from TRANSFAC Professional 2011.2^58^ were screened for enrichment in cell-type-specific hypersensitive regions, in both orientations and with half-site spacing up to 50 bp. Reported *p*-values were Bonferroni-corrected in a conservative approach, accounting for all possible motif complexes that could be formed by 964 vertebrate motifs available in TRANSFAC.

### Protein production

The 79aa HMG boxes SOX4, SOX6, SOX8, SOX9, SOX10 (with N-terminal His6-tag), SOX18 and the 109aa SOX2 HMG box (without N-terminal His6-tag) were prepared as described^47^,^59^. Constructs containing the DIM domain termed SOX9-DHMG and SOX10-DHMG and constructs starting at the N-terminal ATG and ending after the HMG box termed SOX8-NHMG and SOX9-NHMG were cloned into pETG60A or pDEST-hisMBP expression plasmids using the Gateway BP and LR cloning system (Invitrogen) using primers listed in Table 1. Proteins were expressed in BL21(DE3) or BL21(DE3)pLys and grown in Terrific Broth (TB) in the presence of 1 mM IPTG at 18 °C for 18 h. Cells were pelleted and resuspended in lysis buffer (20 mM Tris-HCl pH 8.0; 300 mM NaCl; 20 mM Imidazole) and lysed by high-pressure disruption (Guangzhou JuNeng Biology & Technology , JN3000 PLUS) at 4 °C. Fusing protein were extracted from cellular lysates using Ni-NTA Agarose (Qiagen), eluted with elution buffer (20 mM Tris-HCl pH 8.0; 100 mM NaCl; 300 mM Imidazole) and then cleaved by adding 1/30 (weight per weight) tobacco etch virus (TEV) protease at 4 °C for 24 h. SOXE constructs were then purified using a 6 mL Resource S column (GE Healthcare) connected to an AKTAxpress system (GE Healthcare) with a NaCl gradient from 100 mM to 1M. Proteins were desalted using PD-10 columns (GE healthcare) and a buffer containing 20 mM Tris-HCl pH 8.0; 100 mM NaCl. The concentration was determined by measuring the absorbance at 280 nm with a Nanodrop 2000 spectrophotometer and proteins were aliquoted and stored at −80 °C.

### Electrophoretic mobility shift assay

DNA oligos were procured from Life Technologies and dsDNA probes were generated by mixing cy5-labelled forward and unlabeled reverse strands in 1X annealing buffer (20 mM Tris–HCl, pH 8.0; 50 mM MgCl2; 50 mM KCl) and heating to 95 °C for 5 min and subsequent cooling to 4 °C at with 1 °C /min in a PCR block. Each EMSA reaction was carried out using a 1X EMSA buffer (10  mM Tris–HCl pH 8.0, 0.1 mg/ml bovine serum albumin, 50 μM ZnCl2, 100 mM KCl, 10% (v/v) glycerol, 0.1% (v/v) Igepal CA630 and 2 mM beta-mercaptoethanol), 50 nM dsDNA and varying protein concentrations for 4 h at 4 °C in the dark. For heterodimer assays the 4bp spacer probe was selected. After incubation, samples were loaded onto 12% 1X Tris-glycine (25 mM Tris-HCl pH 8.0, 192 mM glycine) native PAGE gels and run at 200 V for 35–40 min in 1X TG buffer in the cold room. Bands were visualized using a Typhoon FLA-7000 PhosphorImager (FUJIFILM) and quantified using the Image Quant software (GE Healthcare). Cooperativity factors were calculated as described previously^36^.

### Circular Dichroism

For circular dichroism spectra, 5 μM of SOX9-HMG, SOX9-DHMG and SOX8-NHMG proteins and 1 μM of pre-annealed un-labeled dsDNA (5’-CCGaacaatgGAAGcattgttGCC-3’) were used. Samples were incubated at 4 °C for 4 h in 50 mM phosphate buffer, pH 8.0 before measurements. Spectra were recorded on an Chirascan CD Spectrometer (Applied Photophysics Ltd) with a strain-free 10 mm × 1.0 mm rectangular cuvette and at a 1 nm bandwidth, spectral range, 180–320 nm; step-size, 1 nm; time-pep-point, 0.5 s.

## Additional Information

**How to cite this article**: Huang, Y.-H. *et al*. SOXE transcription factors form selective dimers on non-compact DNA motifs through multifaceted interactions between dimerization and high-mobility group domains. *Sci. Rep.* **5**, 10398; doi: 10.1038/srep10398 (2015).

## Figures and Tables

**Table 1:** Primers used to produce expression constructs for SOXE proteins.

**Primer name**	**Sequence**
SOX8_NHMG_F	GGGGACAAGTTTGTACAAAAAAGCAGGCTTCGAAAACCTGTATTTTCAGGGCatgCTGGACATGAGTGAGGC
SOX8_DHMG_F	GGGGACAAGTTTGTACAAAAAAGCAGGCTTCGAAAACCTGTATTTTCAGGGCgatACTGCTGAGGCAGCAGACG
SOX8_HMG_R	GGGGACCACTTTGTACAAGAAAGCTGGGTTTATCAactCTTCCTTCGCCTTGGCTG
SOX9_DHMG_F	GGGGACAAGTTTGTACAAAAAAGCAGGCTTCGAAAACCTGTATTTTCAGGGCctgAAGAAGGAGAGCGAGGAAG
SOX9_HMG_R	GGGGACCACTTTGTACAAGAAAGCTGGGTTTATCAcgaCTTCCTCCGCCGG
SOX10_NHMG_F	GGGGACAAGTTTGTACAAAAAAGCAGGCTTCGAAAACCTGTATTTTCAGGGCatgGCCGAGGAACAAGACC
SOX10_DHMG_F	GGGGACAAGTTTGTACAAAAAAGCAGGCTTCGAAAACCTGTATTTTCAGGGCggcGAGGCGGACGATGACAAG
SOX10_HMG_R	GGGGACCACTTTGTACAAGAAAGCTGGGTTTATCAgttCTTCCGCCGCCGAGGTT

‘F’ and ‘R’ denote forward and reverse primers. First and last codons of the gene specific region are in small caps.
